# The Chromatin of *Candida albicans* Pericentromeres Bears Features of Both Euchromatin and Heterochromatin

**DOI:** 10.3389/fmicb.2016.00759

**Published:** 2016-05-19

**Authors:** Verónica Freire-Benéitez, R. Jordan Price, Alessia Buscaino

**Affiliations:** School of Biosciences Canterbury Kent, University of KentCanterbury, UK

**Keywords:** epigenetics, chromatin, centromere, *Candida albicans*, heterochromatin

## Abstract

Centromeres, sites of kinetochore assembly, are important for chromosome stability and integrity. Most eukaryotes have regional centromeres epigenetically specified by the presence of the histone H3 variant CENP-A. CENP-A chromatin is often surrounded by pericentromeric regions packaged into transcriptionally silent heterochromatin. *Candida albicans*, the most common human fungal pathogen, possesses small regional centromeres assembled into CENP-A chromatin. The chromatin state of *C. albicans* pericentromeric regions is unknown. Here, for the first time, we address this question. We find that *C. albicans* pericentromeres are assembled into an intermediate chromatin state bearing features of both euchromatin and heterochromatin. Pericentromeric chromatin is associated with nucleosomes that are highly acetylated, as found in euchromatic regions of the genome; and hypomethylated on H3K4, as found in heterochromatin. This intermediate chromatin state is inhibitory to transcription and partially represses expression of proximal genes and inserted marker genes. Our analysis identifies a new chromatin state associated with pericentromeric regions.

## Introduction

The centromere is the *cis*-acting DNA site of kinetochore assembly and spindle attachment during chromosome segregation in mitosis and meiosis. Centromeric regions have a different organization across different species. Some organisms, such as the budding yeast *Saccharomyces cerevisiae*, have “point” centromeres while other organisms, such as the fission yeast, the fruit fly and human, have “regional” centromeres (Buscaino et al., 2010). Point centromeres are only ~125 bp long and include specific DNA binding sites necessary for centromere function (Westermann et al., 2007). Regional centromeres span large DNA domains (~10 to 10,000 kb) and do not contain a specific DNA sequence but are epigenetically specified by the presence of the histone H3 variant, CENP-A (also termed Cse4 and CENH3) (Buscaino et al., 2010). Regional centromeres are often associated with repetitive elements. The structure and organization of centromere-associated DNA repeats varies across organisms. For example, human centromeres are composed of tandem arrays of 171 alpha-satellite repeats and, in *Drosophila melanogaster*, centromeric DNA contains short repetitive elements interspersed with transposable elements (Buscaino et al., 2010). In the yeast *Schizosaccharomyces pombe* and the fungal pathogen *Candida tropicalis*, centromeres are organized in a CENP-A-containing central/mid core domain flanked by inverted repeats (IRs) whose sequences are conserved across centromeres (Buscaino et al., 2010; Chatterjee et al., 2016). In both organisms, these IRs are important for *de novo* CENP-A deposition on a plasmid containing the central core sequence (Baum et al., 1994; Chatterjee et al., 2016). Pericentromeric regions are usually assembled into transcriptionally silent heterochromatin that is required for establishment of CENP-A chromatin and for faithful chromosome segregation (Bernard et al., 2001; Nonaka et al., 2002; Folco et al., 2008). At these locations, heterochromatin is hypoacetylated at Lysine 9 of Histone H3 (H3K9) and Lysine 16 of Histone H4 (H4K16). Heterochromatic regions are also hypomethylated at Lysine 4 of Histone H4 (H3K4) and methylated at H3K9 (Strahl and Allis, 2000; Kouzarides, 2007). Histone modifiers control this modification state: for example the histone deacetylase (HDAC) Sir2 deacetylates H3K9 and/or H4K16, the histone methyltransferase Set1 methylates H3K4 and the histone methyltransferase Su(var)3-9 methylates H3K9 (Shankaranarayana et al., 2003; Wirén et al., 2005; Bühler and Gasser, 2009; Kueng et al., 2013). Although pericentromeric heterochromatin is usually associated with pericentromeric regions, this repressive chromatin state is not absolutely required for centromere function and faithful chromosome segregation. For example, in *Candida lusitaniae* pericentromeric regions are not assembled into heterochromatin (Kapoor et al., 2015). Given the diversity of centromere structure across eukaryotes, it is important to analyze centromere organization in a variety of organisms.

**FIGURE 1:**
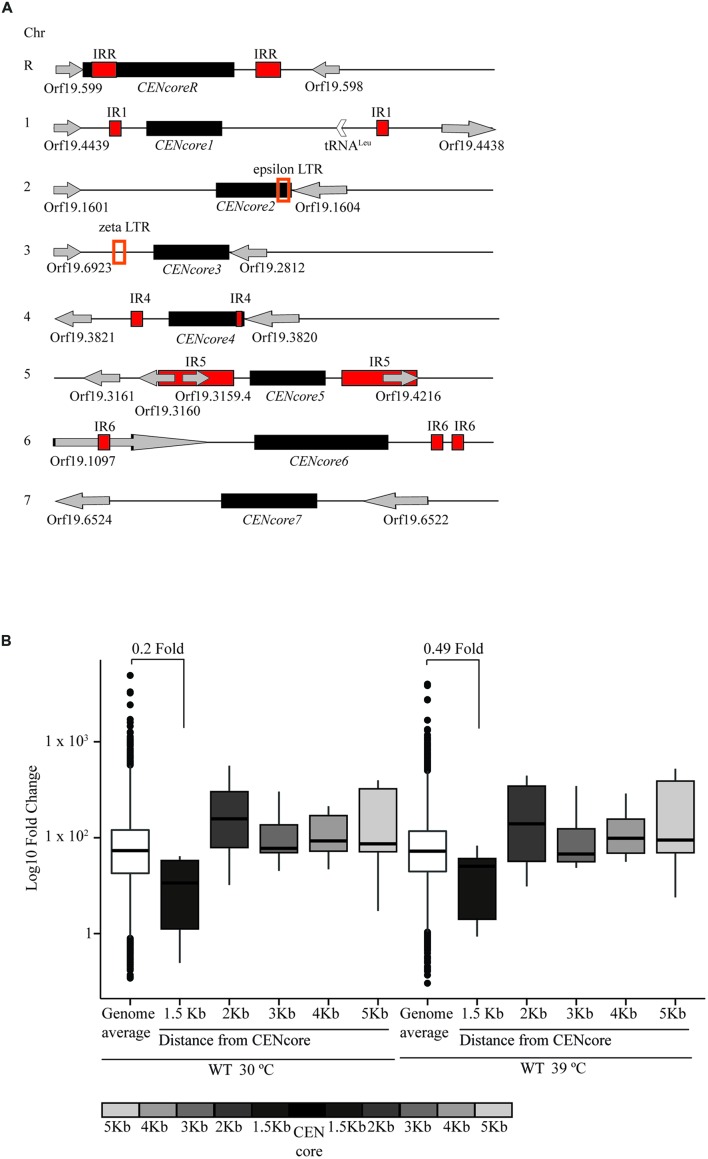
**Genes flanking centromeres display reduced expression. (A)** Schematic of *Candida albicans* centromeres. On each chromosome, black squares indicate centromere core regions (*CENcore*). Red squares indicate inverted repeats (IRs). Orange empty squares indicate Long Terminal Repeats (LTRs). Empty arrows indicate tRNA codons. Gray arrows indicate ORF and their transcription orientation. **(B)** RNA deep-sequencing of WT isolates at 30°C and 39°C. Boxplots represent normalized read counts (FPKM) of median genome expression compare to the median of all *CENcore* proximal genes from 1.5 to 5 Kb flanking both sides of *CENcore* regions on all chromosomes.

*Candida albicans*, the most common human fungal pathogen, is an ideal system to investigate diversity and structure of centromeres because it possess regional centromeres that are much smaller and simpler than other regional centromeres (Sanyal et al., 2004; Baum et al., 2006; Mishra et al., 2007). Each of the 8 *C. albicans* diploid chromosomes has a relatively small regional centromere (2–4 kb) assembled into CENP-A chromatin (Baum et al., 2006). The organization and sequence of pericentromeric regions differs at each centromere (**Figure 1A**). Centromeres on chromosome 1, 4, 5, 6, and R are similar to centromeres of the fission yeast *Schizosaccharomyces pombe* where IRs flank the CENP-A containing domain. Contrary to *S. pombe*, the sequence of these repetitive elements is not conserved across centromeres. On chromosome 2 and 3 Long Terminal Repeats (LTRs) are found within ~3 kb of the CENP-A containing domain. Centromere on chromosome 7 does not have obvious repeats nearby (Mishra et al., 2007; Ketel et al., 2009). Therefore, 7 of the 8 pericentromeric regions are associated with DNA repeats. Several lines of evidence suggest that, in *C. albicans*, pericentromeric repeats are important for centromere function and/or establishing centromere identity. First of all, despite the lack of conservation in the DNA sequence, repetitive DNA is also associated with centromere of other *Candida* species such as *C. dubliniensis* (Padmanabhan et al., 2008). In addition, following deletions of endogenous centromeres, *C. albicans* neocentromeres form efficiently and are often assembled in proximity to DNA repeats (Ketel et al., 2009). However, the lack of repetitive elements surrounding the centromere on chromosome 7 argue against a role of DNA repeats in centromere function. In many eukaryotes, pericentromeric regions are assembled into transcriptionally silent heterochromatin. It is possible that, despite the lack of a conserved DNA sequence and/or DNA feature, the common feature of *C. albicans* pericentromeric regions is a specific chromatin structure resembling heterochromatin. Here, we address this question.

The *C. albicans* epigenome, similarly to *S. cerevisiae*, lacks Su(var) 3–9 orthologs, hence H3 K9 methylation is absent in both *C. albicans* and *S. cerevisiae.*

We have recently shown that, in *C. albicans*, transcriptionally silent heterochromatin is assembled at the ribosomal DNA (*rDNA*) locus and at telomeres (Freire-Benéitez et al., 2016). At these locations, heterochromatin is typified by nucleosomes that are hypoacetyled and hypomethylated on H3K4. The histone deacetylateses Sir2 (*orf 19.1992*) is required to maintain this repressive epigenetic state via hypoacetylation of H3K9 and H4K16 (Freire-Benéitez et al., 2016).

In this study, we investigate the chromatin state associated with *C. albicans* pericentromeric repeats. We find that pericentromeric regions are assembled into an intermediate chromatin state bearing features of both euchromatin (high histone acetylation) and heterochromatin (hypomethylation of H3K4). This intermediate chromatin state is associated with a weak transcriptionally silent environment that partially represses expression of proximal genes and inserted marker genes. Our analysis identifies a new chromatin state associated with pericentromeric regions.

## Materials and Methods

### Growth Conditions

Yeast cells were cultured in rich medium (YPAD) containing extra adenine (0.1 mg/ml) and extra uridine (0.08 mg/ml), complete SC medium (Formedium^TM^), or SC Drop-Out media (Formedium^TM^). Cells were grown at 30 or 39°C as indicated.

### Yeast Strain Construction

Strains are listed in Supplementary Table S1. Integration and deletion of genes were performed using plasmids containing marker genes for substitution or integration at endogenous locus as previously described (Wilson et al., 1999). Transformation was performed by electroporation (Gene Pulser^TM^, Bio-Rad) using the protocol described in (De Backer et al., 1999). *URA3* marker gene was used for silencing assays to determine *URA3* expression in complete SC medium (Formedium^TM^) or SC URA Drop-Out media (Formedium^TM^). *HIS1*, *ARG4*, and *NAT* marker genes were used to replace both copies of *SIR2*, *SET1*, and *JHD2* genes. PCR was used for screening of positive transformants. Oligonucleotides and plasmids used for strain constructions are listed in Supplementary Tables S2 and S3, respectively.

### Silencing Assay

Growth analyses were performed using a plate reader (SpectrostarNano, BMG labtech) in 96 well plate formats at 30°C. For each silencing assay in 96 well plate format, 1:100 dilution of and overnight culture was inoculated in a final volume of 95 μl of SC or SC-URA media to reach a concentration of 60 cells/μl. Growth was assessed by measuring A_600_, using the following conditions: OD_600 nm_, 616 cycle time, three flashes per well, 700 rpm shaking frequency, orbital shaking mode, 545 s additional shaking time after each cycle 0.5 s post delay, for 44 h. Graphs represent data from three biological replicates. Error bars: standard deviation (SD) of three biological replicates generated from three independent cultures of the same strain. Data was processed using SpectrostarNano MARS software and Microsoft Excel.

### RNA Extraction and cDNA Synthesis

RNA was extracted from Log2 exponential cultures (OD_600 nm_ = 1.4) using a yeast RNA extraction kit (E.Z.N.A. ^®^Isolation Kit RNA Yeast, Omega Bio-Tek) following the manufacturer’s instructions. RNA quality was checked by electrophoresis under denaturing conditions in 1% agarose, 1X HEPES, 6% Formaldehyde (Sigma). RNA concentration was measured using a NanoDrop ND-1000 Spectrophotometer. cDNA synthesis was performed using iScript^TM^ Reverse Transcription Supermix for RT-qPCR (Bio-Rad) following manufacturer’s instructions and a Bio-Rad CFXConnect^TM^ Real-Time System.

### High-throughput RNA Sequencing

Strand-specific cDNA Illumina Barcoded Libraries were generated from 1 μg of total RNA extracted from wt and *sir2*Δ/Δ strains and sequenced with an Illumina iSeq2000 platform. Illumina Library and Deep-sequencing was performed by the Genomics Core Facility at EMBL (Heidelberg, Germany). Raw reads were analyzed using TopHat algorithm following the RNA deep sequencing analysis pipeline described (Trapnell et al., 2013) using Galaxy^1^ and Linux platform. Heatmaps and boxplot graphs were generated with R^2^. RNA sequencing data are deposited into ArrayExpress (accession number E-MTAB-4622).

### Quantitative Chromatin ImmunoPrecipitation (qChIP)

Quantitative Chromatin ImmunoPrecipitation (qChIP) was performed as described (Pidoux et al., 2004) with the following modifications: 5 ml of an overnight culture grown in YPAD with extra uridine (0.08 mg/ml), diluted into fresh YPAD with extra uridine (0.08 mg/ml) and grown until OD600 nm of 1.4. Cells (50 ml/sample) were fixed with 1% Paraformaldehyde (Sigma) for 15 min at room temperature. Cells were lysed using acid-washed glass beads (Sigma) and a Disruptor genie^TM^ (Scientific Industries) for 30 min at 4°C. Chromatin was sheared to 500–1000 bp using a Bioruptor (Diagenode) for a total of 20 min (30 s ON and OFF cycle) at 4°C. Immunoprecipitation was performed overnight at 4°C using 2 μL of antibody anti-H3K4me2 (Active Motif- Cat Number: 39141), anti-H3K9ac (Active Motif- Cat Number: 39137), and anti-H4K16ac (Active Motif- Cat Number: 39167) and 25 μl of Protein G magnetic beads (Dynal – InVitrogen). DNA was eluted with a 10% slurry of Chelex 100-resin (Bio-Rad) using the manufacturer’s instructions.

### qPCR Reactions

Primers used are listed in Supplementary Table S2. Real-time qPCR and RT-qPCR were performed in the presence of SYBR Green (Bio-Rad) on a Bio-Rad CFXConnect^TM^ Real-Time System. Data was analyzed with Bio-Rad CFX Manager 3.1 software and Microsoft Excel. Enrichments were calculated as the percentage ratio of specific IP over input for qChIP analysis and as enrichment over actin for RT-qPCR. Histograms represent data from three biological replicates. Error bars: SD of three biological replicates generated from three independent cultures of the same strain.

## Results

### Expression Level of Centromere-Proximal Genes Is Low

Heterochromatin represses expression of associated and proximal genes (Freitas-Junior et al., 2005; Kaur et al., 2005; Hansen et al., 2006; Merrick and Duraisingh, 2006). Therefore, if the pericentromeric regions of *C. albicans* were assembled into heterochromatin, genes in proximity to these regions would be poorly expressed. To test this hypothesis, we isolated RNA from wild-type (WT) cells grown at a temperature relevant for growth of *C. albicans* on the skin (30°C) and at a temperature mimicking fever in the host (39°C) and performed RNA-seq analyses. FPKM (fragments per kilobase of exons per million mapped reads) values were determined for each annotated gene. We then calculated the FPKM for genes in cumulative bins of 1 kb from the centromeres and compared these values to the genome-wide average (**Figure 1B**). This analysis reveals that genes in proximity to centromeres (0 to 1.5 kb) are less expressed compared with the genome-wide average (*p*-value = 2.2 × 10^-16^). These data suggest that pericentromeric and centromeric regions impose a weak transcriptionally repressive environment.

### A Marker Gene Inserted at Pericentromeric Repeats Is Partially Repressed

**FIGURE 2:**
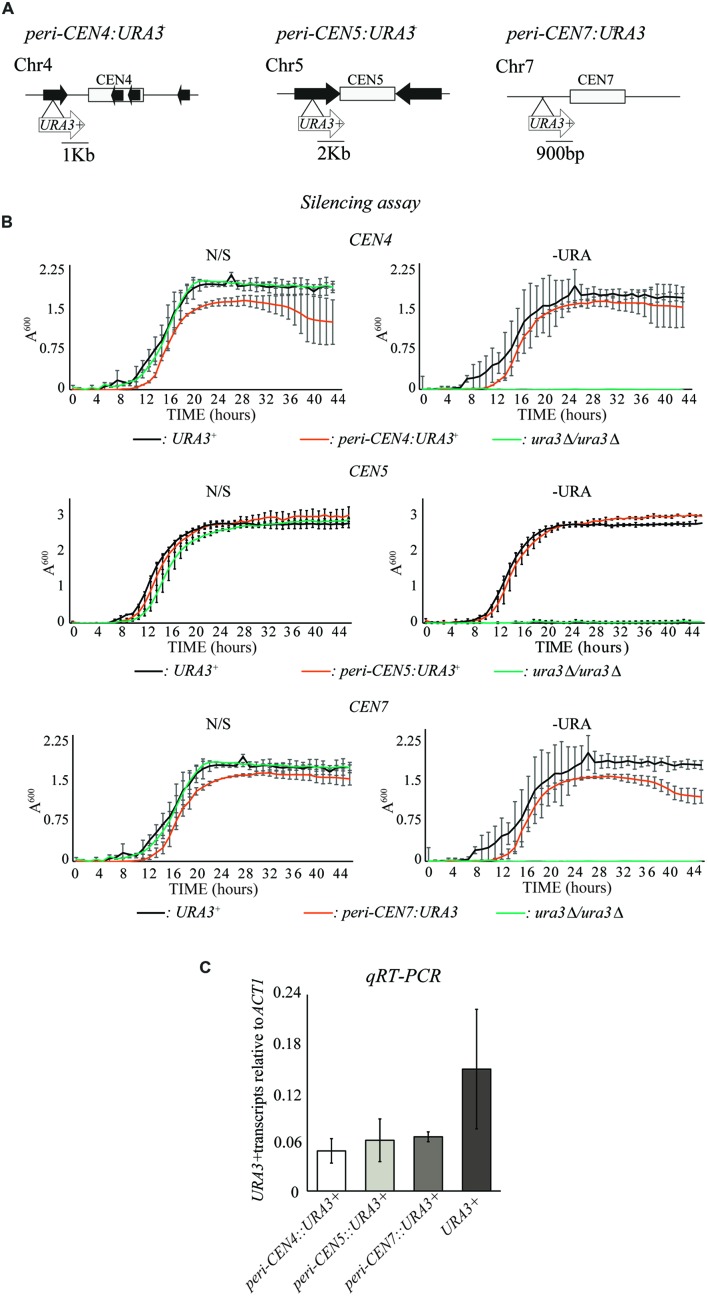
**Weak transcriptional silencing of a URA3 marker gene integrated at pericentromeric region. (A)** Schematic of *peri-CEN4:URA3*^+^, *peri-CEN5:URA3*^+^, and peri-*CEN7:URA3*^+^ reporter strains. Distance from the *URA3* marker gene to each *CENcore* is indicated in Kb **(B)** Silencing assay of the *peri-CEN4:URA3*^+^, *peri-CEN5:URA3*^+^, and *peri*-*CEN7:URA3*^+^ reporter strain. *URA3*^+^ (*URA3/URA3)* and Ura^-^ (*ura3*Δ*/ura3*Δ) strains were included as controls. **(C)** *qRT-PCR* analyses to measure *URA3*^+^ transcript levels of the *peri-CEN4:URA3*^+^, *peri-CEN5:URA3*^+^, and *peri*-*CEN7:URA3*^+^ reporter strain relative to actin transcript levels (*ACT1*). Error bars in each panel: standard deviation (SD) of three biological replicates.

Heterochromatin assembled onto repetitive DNA represses the transcription of marker genes inserted in their proximity (Henikoff and Dreesen, 1989; Gottschling et al., 1990; Bryk et al., 1997; Smith and Boeke, 1997). We have previously shown that, also in *C. albicans*, heterochromatin silences inserted marker genes (Freire-Benéitez et al., 2016). Our RNA-seq analyses suggest that regions proximal to a centromere impose a weak transcriptional repressive environment (**Figure 1B**). To test whether *C. albicans* pericentromeres impose transcriptional silencing dependently or independently of the presence of DNA repeats, we integrated the URA3 marker gene into the pericentromeric regions of centromeres surrounded by DNA repeats (*peri-CEN4:URA3*^+^ and *peri-CEN5:URA3*^+^) and into the pericentromeric regions of CEN7, lacking DNA repeats (*peri-CEN7:URA3*^+^). (**Figure 2A**). To investigate whether the URA3 marker gene is transcriptionally silenced when inserted at these locations, strains were grown in non-selective (N/S) medium and in medium lacking uridine (–Uri) in which only cells expressing sufficient Ura3 protein are able to grow. Silencing of *URA3* is expected to result in slower growth in –Uri medium compared to N/S. However, none of the strains grew poorly in –Uri medium compared to N/S medium (**Figure 2B**). In contrast, quantitative reverse transcriptase analysis (*qRT-PCR*) reveals that the levels of *URA3* mRNA for the *peri-CEN4:URA3*^+^*, peri-CEN5:URA3*^+^, and *peri-CEN7:URA3*^+^ strains were significantly lower than the *URA3* euchromatic gene (**Figure 2C**). Therefore, pericentromeric regions impose a weak transcriptional silencing that can only be detected at the RNA level independently of the presence of repetitive elements.

### Pericentromeric Repeats Are Assembled Into an Intermediate Chromatin State Bearing Features of Both Euchromatin and Heterochromatin

**FIGURE 3:**
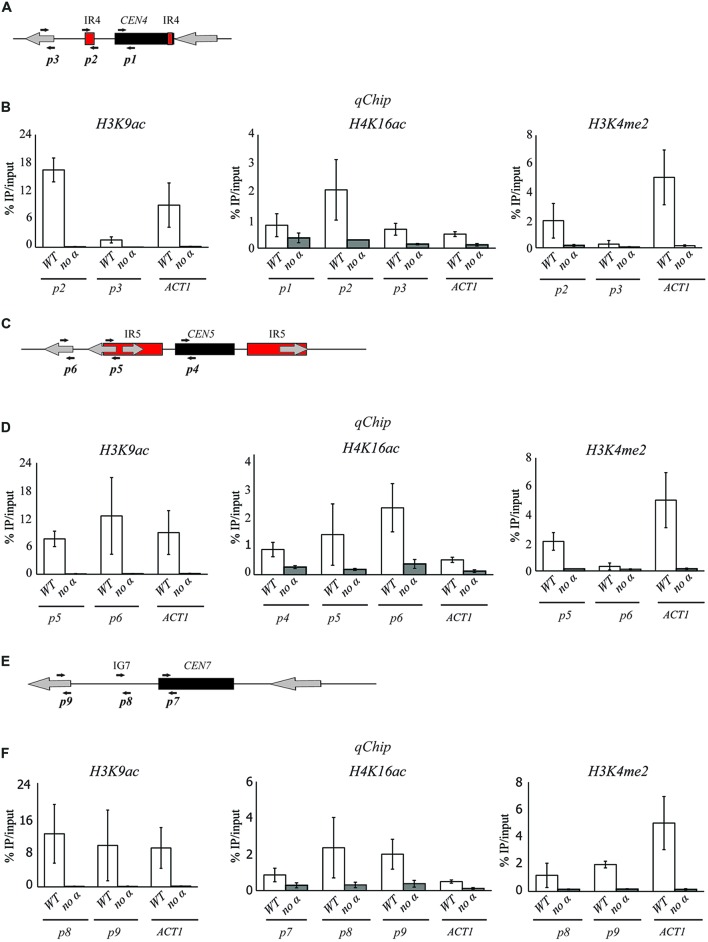
**The chromatin state of *C. albic*ans centromeres. (A)** Schematic of *C. albicans CEN4*. Black arrows indicate the primers used to study the chromatin pattern associated with *CEN4* **(B)** *qChIP* to detect H3K9ac and H3K4me2 levels associated with CEN 4. *ACT1* was used as a control. **(C)** Schematic of *C. albicans CEN5*. Black arrows indicate the primers used to study the chromatin pattern associated with *CEN5*. **(D)** *qChIP* to detect H3K9ac and H3K4me2 levels associated with CEN5. *ACT1* was used as a control. **(E)** Schematic of *C. albicans CEN7*. Black arrows indicate the primers used to study the chromatin pattern associated with *CEN7*. **(F)** *qChIP* to detect H3K9ac, H4K16ac and H3K4me2 levels associated with *CEN7*. *ACT1* was used as a control. Error bars in each panel: SD of three biological replicates.

We have shown that, in *C. albicans*, heterochromatin regions are assembled into nucleosomes that are hypoacetylated on H3K9 and H4K16 and hypomethylated on H3K4 (Freire-Benéitez et al., 2016). To assess whether pericentromeric regions are associated with heterochromatic histone marks, we monitored by *qChIP* the presence of H3K9Ac, H4K16Ac, and H3K4me at pericentromeric regions surrounding CEN4, CEN5, and CEN7 (**Figure 3A**). As a control, the chromatin state associated with the euchromatic *ACT1* locus was analyzed. We find that all pericentromeric regions analyzed are assembled into chromatin that is highly acetylated on H3K9 and H4K16 as levels of these two histone modifications are similar to levels detected at the active and euchromatic locus *ACT1* (**Figures 3B,D**, and **F**). High levels of H4K16 acetylation are also found at the central core region assembled into CENP-A chromatin (**Figure 3**). In contrast, pericentromeric chromatin is hypomethylated on H3K4, a chromatin state more similar to heterochromatic regions and different from the euchromatic *ACT1* locus (**Figures 3B,D**, and **F**). Thus, pericentromeric regions have only one of the three marks (H3K4 hypomethylation) associated with classic heterochromatin. Importantly, this chromatin state marks pericentromeric regions independently of the presence of repeats as pericentromeres with DNA repeats (CEN4 and CEN5) and without (CEN7) are associated with a similar histone modifications pattern. We concluded that *C. albicans* pericentromeres are not assembled into classical transcriptionally silent heterochromatin but they are associated with an intermediate chromatin state bearing features of euchromatin (high histone acetylation) and heterochromatin (H3K4 hypomethylation).

### The Chromatin State Associated with Pericentromeric Regions Is Independent of the Histone Deacetylase Sir2

**FIGURE 4:**
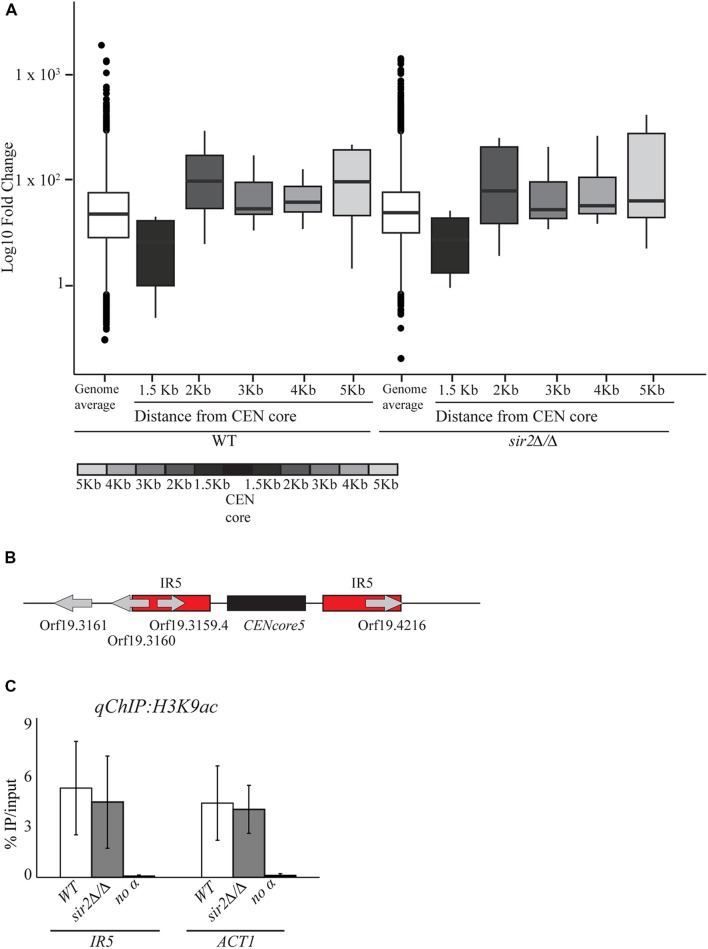
**The chromatin state associated with *C. albicans* centromeres is independent of the histone deacetylase (HDAC) Sir2. (A)** RNA deep-sequencing of WT and *sir2*Δ/Δ. Boxplots represent normalized read counts (FPKM) of median genome expression compare to the median of all *CEN* proximal genes from 1.5 to 5 Kb **(B)** Schematic of *C. albicans CEN5*. Black arrows indicate the primers used to study the chromatin pattern associated with the IRs5 of *CEN5*. **(C)** *qChIP* to detect H3K9ac levels associated with the IRs5 of *CEN5* and *ACT1* in WT and *sir2* Δ/Δ strains. Error bars in each panel: SD of three biological replicates.

The HDAC Sir2 specifically deacetylates H3K9 and H4K16 and it is required for heterochromatin assembly across the eukaryotic kingdom (Rusche et al., 2003). We have shown that *C. albicans* Sir2 is necessary for heterochromatin integrity at the *rDNA* locus and telomeric regions via deacetylation of H3K9 and H4K16 (Freire-Benéitez et al., 2016). To assess whether Sir2 contributes to the chromatin and transcriptional state of pericentromeric regions, we isolated RNA from WT and *sir2* Δ/Δ cells and performed RNA-seq analyses. FPKM values were determined for all genes proximal to CEN repeats and compared between *sir2* Δ/Δ and WT strains. Upon deletion of the *SIR2* gene, we did not observe any clear effect on expression of *CEN* proximal genes (**Figure 4A**). Only 2 out of the 12 genes located in proximity (<1.5 Kb) of centromeres were expressed more than 2 fold in *sir2* Δ/Δ isolates compared to WT cells (**Table 1**). Therefore Sir2 does not contribute to the poor expression of *CEN*-proximal genes. In agreement with these results, deletion of the *SIR2* gene does not increase the levels of H3K9 and H4K16 acetylation associated with pericentromeric region on chromosome five as revealed by *q-ChIP* analyses (**Figure 4C**).

**Table 1:** RNA deep-sequencing values of 1.5 Kb CEN proximal genes.

ORF	Gene	CENcore	bp distance to CENcore	*WT* FPKM	*sir2*Δ/Δ FPKM	(*sir2Δ/Δ/WT)* FPKM Fold	Log2(Fold)
*orf19.4439*	-	1	1500	6.60168	10.8237	1.639537	0.713284
*orf19.1604*	-	2	96	27.1566	36.3803	1.339649	0.421855
*orf19.2812*	-	3	100	29.8328	14.1075	0.472886	-1.08044
*orf19.3820*	-	4	51	0.913477	0.96385	1.055144	0.07744
*orf19.3159.4*	-	5	926	0.516172	2.34077	4.534864	2.18106
*orf19.1097*	*ALS2*	6	1498	9.68354	39.5448	4.083713	2.029882
*orf19.2123*	-	6	231	34.9161	47.2585	1.353487	0.43668
*orf19.2124*	-	6	765	20.2353	29.1543	1.440764	0.526838
*orf19.599*	-	R	102	1.24606	1.76585	1.417147	0.502991
*orf19.598*	-	R	1126	0.209874	1.269	6.046485	-1.16192

*ORFs include all genes within 1.5 Kb of a centromere core. Values are indicated in FPKM for WT and sir2 Δ/Δ strains, including FPKM fold increase and Log2 fold increase of sir2 Δ/Δ strain over WT strain*.

### The Histone Methyltransferase Set1, But Not the Histone Demethylase Jhd2, Contributes to the Chromatin State Associated with Pericentromeres

**FIGURE 5:**
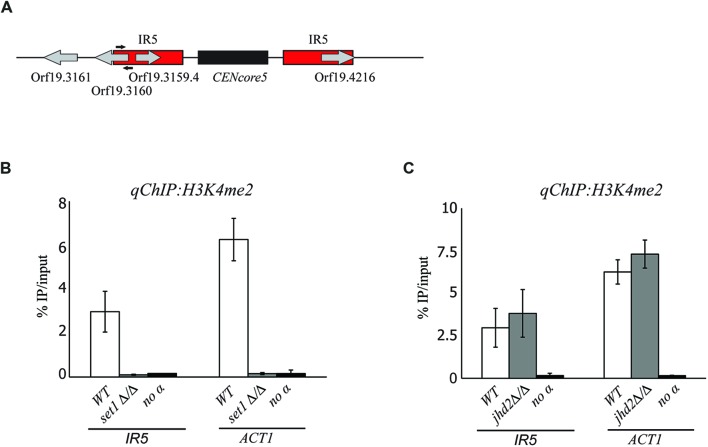
**The chromatin state associated with pericentromeric regions is independent of the histone methyltransferase Set1 and the histone demethylase Jhd2. (A)** Schematic of *C. albicans CEN5.* Black arrows indicate the primers used to study the chromatin pattern associated with the IRs5 of *CEN5*. **(B)** *qChIP* to detect H3K4me2 levels associated with the IRs5 of *CEN5* and *ACT1* in WT and *set1* Δ/Δ strains. **(C)** *qChIP* to detect H3K4me2 levels associated with the IRs5 of *CEN5* and *ACT1* in WT and *jhd2 Δ/Δ* strains. Error bars in each panel: SD of three biological replicates.

Coordination of activities between histone methyltransferases and histone demethylases ensures the right methylation level associated with euchromatic and heterochromatic loci. Therefore, specific histone methyltransferases and demethylases might be important for maintaining the H3K4 hypomethylated state associated with *C. albicans* pericentromeres. The *C. albicans* genome encodes for the H3K4 methyltransferase Set1 (Raman et al., 2006) and for the putative H3K4 demethylase Kmd5/Jhd2 (*orf19.5651*). In *S. cerevisiae*, both proteins have been implicated in transcriptional silencing and heterochromatin formation (Briggs et al., 2001; Ingvarsdottir et al., 2007; Ryu and Ahn, 2014). To assess whether Set1 and/or Jhd2 contribute to the chromatin state associated with *C. albicans* pericentromeric regions, we deleted both copies of the *SET1* and *JHD2* genes from WT cells and quantified H3K4me2 levels by *qChIP* analyses. We find that Set1 is necessary for maintaining the low levels of H3K4me associated with *CEN5* repeats (**Figure 5A**) as H3K4me dropped to background levels in *set1*Δ/Δ compared to WT cells (**Figure 5B**). In contrast, we find that Jhd2 is not required for maintaining the hypomethylated state associated with *CEN5* (**Figure 5A**) repeats as H3K4me levels did not change between *jhd2*Δ/Δ and WT cells (**Figure 5C**).

## Discussion

This study is the first analysis of the chromatin state associated with pericentromeric regions in the human fungal pathogen *C. albicans*.

In many organisms, regional centromeres have a conserved modular structure despite the lack of a conserved DNA sequence. At these locations, CENP-A domains, sites of kinetochore assembly, are flanked or interspersed by DNA repeats assembled into heterochromatin (Buscaino et al., 2010). Both CENP-A chromatin and pericentromeric heterochromatin are inhibitory to transcription as illustrated by silencing of inserted marker genes (Allshire et al., 1994; Ketel et al., 2009).

The small *C. albicans* regional centromeres are composed of a CENP-A central core surrounded by pericentromeric regions with different sequence and organization. CENP-A chromatin represses transcription of embedded marker genes (Ketel et al., 2009). Here, we show that, despite the absence of canonical heterochromatin, *C. albicans* pericentromeric regions weakly repress transcription. These conclusions are supported by two observations. Firstly, RNA-sequence analysis highlights that genes located in proximity (<1.5 Kb) to centromeres are less expressed than the genome-wide average (**Figure 1B**). Secondly, a *URA3* marker gene inserted into the pericentromeric region of chromosomes 4, 5, and 7 is weakly silenced. This weak silencing is observed when *URA3* RNA levels are measured by *qRT-PCR* but no silencing is detected by growing strains in –URA medium (**Figure 2**). This suggests that the reduced expression of the *URA3*^+^ marker gene is sufficient to confer a URA^+^ growth phenotype.

*Candida albicans* lacks the heterochromatic structure that is normally associated with regional centromeres because the *C. albicans* genome does not encode for *SuVar* 3–9, the H3K9-specific methyltransferase, or for HP-1, the chromodomain protein responsible for assembly and spreading of centromeric heterochromatin. We have recently shown that transcriptionally silenced heterochromatin exists in *C. albicans* and it is associated with the *rDNA* locus and telomeric regions (Freire-Benéitez et al., 2016). This heterochromatic state, similarly to *S. cerevisiae*, is characterized by hypoacetylated nucleosomes that are hypomethylated on H3K4 (Freire-Benéitez et al., 2016). Here, we show that *C. albicans* pericentromeric regions are not associated with heterochromatin but with an intermediate chromatin state bearing features of both euchromatin and heterochromatin. Nucleosomes at pericentromeric regions are highly acetylated, as observed in euchromatin, and hypomethylated on H3K4, as observed in heterochromatin (**Figure 3**). Consistently, we find that deletion of the HDAC Sir2 does not perturb the chromatin state of pericentromeric regions (**Figure 4**).

Given the lack of canonical heterochromatin at pericentromeric regions, it is still unclear what drives the weak transcriptional silencing associated with these regions. We envisage two possible scenarios. It is possible that low levels of CENP-A, undetectable by ChIP analyses, associate with pericentromeric regions driving transcriptional silencing. In support of this hypothesis, it is well known that CENP-A chromatin is inhibitory to transcription. In addition, it has been observed that, following deletion of endogenous centromeric sequences, neocentromeres often form immediately adjacent to the site of the excised native centromeres (Ketel et al., 2009; Shang et al., 2013; Thakur and Sanyal, 2013). These data suggest that low levels of CENP-A might be present at pericentromeric regions and might be sufficient to nucleate new centromeres. In agreement with this hypothesis, it has been shown that a pool of free CENP-A accessory molecules is present in vicinity of centromeres. These accessory molecules do not nucleate kinetochore assembly but could allow for rapid incorporation of CENP-A in the event of eviction at the centromere (Haase et al., 2013). Therefore, it is possible that CENP-A accessory molecules drive the transcriptional silencing associated with pericentromeric repeats. Finally, it has been shown that, following neocentromere formation, assembly of CENP-A into transcribed regions is sufficient to repress gene expression (Shang et al., 2013).

An alternative hypothesis is that the chromatin state associated with pericentromeric regions is sufficient to drive transcriptional silencing. We find the pericentromeric regions are hypomethylated on H3K4, an epigenetic heterochromatic mark. It is possible that this mark is sufficient to drive transcriptional repression even in the presence of acetylated histones. In support of this hypothesis, it is well established that methylation of H3K4 correlates with gene expression and heterochromatic regions are marked by H3K4 hypomethylation (Fischle et al., 2003; Lachner et al., 2004).

The biology of *C. albicans* DNA repeats, including pericentromeric regions, is still poorly understood. Future studies will reveal whether and how the novel chromatin state associated with these pericentromeric regions controls centromere function and/or identity.

## Conflict of Interest Statement

The authors declare that the research was conducted in the absence of any commercial or financial relationships that could be construed as a potential conflict of interest.

## Footnotes

1 https://usegalaxy.org/

2 http://www.r-project.org/

## References

[B1] Allshire R. C. Javerzat J. P. Redhead N. J. Cranston G. (1994). Position effect variegation at fission yeast centromeres. *Cell* 76 157–169. 10.1016/0092-8674(94)90180-5 8287474

[B2] Baum M. Ngan V. K. Clarke L. (1994). The centromeric K-type repeat and the central core are together sufficient to establish a functional *Schizosaccharomyces pombe* centromere. *Mol. Biol. Cell* 5 747–761. 10.1091/mbc.5.7.747 7812044 PMC301093

[B3] Baum M. Sanyal K. Mishra P. K. Thaler N. Carbon J. (2006). Formation of functional centromeric chromatin is specified epigenetically in *Candida albicans*. *Proc. Natl. Acad. Sci. U.S.A.* 103 14877–14882. 10.1073/pnas.0606958103 17001001 PMC1595444

[B4] Bernard P. Maure J. F. Partridge J. F. Genier S. Javerzat J. P. Allshire R. C. (2001). Requirement of heterochromatin for cohesion at centromeres. *Science* 294 2539–2542. 10.1126/science.1064027 11598266

[B5] Briggs S. D. Bryk M. Strahl B. D. Cheung W. L. Davie J. K. Dent S. Y. (2001). Histone H3 lysine 4 methylation is mediated by Set1 and required for cell growth and rDNA silencing in *Saccharomyces cerevisiae*. *Genes Dev.* 15 3286–3295. 10.1101/gad.940201 11751634 PMC312847

[B6] Bryk M. Banerjee M. Murphy M. Knudsen K. E. Garfinkel D. J. Curcio M. J. (1997). Transcriptional silencing of Ty1 elements in the RDN1 locus of yeast. *Genes Dev.* 11 255–269. 10.1101/gad.11.2.255 9009207

[B7] Bühler M. Gasser S. M. (2009). Silent chromatin at the middle and ends: lessons from yeasts. *EMBO J.* 28 2149–2161. 10.1038/emboj.2009.185 19629038 PMC2722250

[B8] Buscaino A. Allshire R. Pidoux A. (2010). Building centromeres: home sweet home or a nomadic existence? *Curr. Opin. Genet. Dev.* 20 118–126. 10.1016/j.gde.2010.01.006 20206496

[B9] Chatterjee G. Sankaranarayanan S. R. Guin K. Thattikota Y. Padmanabhan S. Siddharthan R. (2016). Repeat-associated fission yeast-like regional centromeres in the ascomycetous budding yeast *Candida tropicalis*. *PLoS Genet.* 12:e1005839 10.1371/journal.pgen.1005839 PMC4741521 26845548

[B10] De Backer M. D. Maes D. Vandoninck S. Logghe M. Contreras R. Luyten W. H. (1999). Transformation of *Candida albicans* by electroporation. *Yeast* 15 1609–1618. 10.1002/(SICI)1097-0061(199911)15:15<1609::AID-YEA485>3.3.CO;2-P 10572258

[B11] Fischle W. Wang Y. Allis C. D. (2003). Histone and chromatin cross-talk. *Curr. Opin. Cell Biol.* 15 172–183. 10.1016/S0955-0674(03)00013-9 12648673

[B12] Folco H. D. Pidoux A. L. Urano T. Allshire R. C. (2008). Heterochromatin and RNAi are required to establish CENP-A chromatin at centromeres. *Science* 319 94–97. 10.1126/science.1150944 18174443 PMC2586718

[B13] Freire-Benéitez V. Price R. J. Tarrant D. Berman J. Buscaino A. (2016). *Candida albicans* repetitive elements display epigenetic diversity and plasticity. *Sci. Rep.* 6 22989 10.1038/srep22989 PMC4789652 26971880

[B14] Freitas-Junior L. H. Hernandez-Rivas R. Ralph S. A. Montiel-Condado D. Ruvalcaba-Salazar O. K. Rojas-Meza A. P. (2005). Telomeric heterochromatin propagation and histone acetylation control mutually exclusive expression of antigenic variation genes in malaria parasites. *Cell* 121 25–36. 10.1016/j.cell.2005.01.037 15820676

[B15] Gottschling D. E. Aparicio O. M. Billington B. L. Zakian V. A. (1990). Position effect at *S. cerevisiae* telomeres: reversible repression of Pol II transcription. *Cell* 63 751–762. 10.1016/0092-8674(90)90141-Z 2225075

[B16] Haase J. Mishra P. K. Stephens A. Haggerty R. Quammen C. Taylor R. M. (2013). A 3D map of the yeast kinetochore reveals the presence of core and accessory centromere-specific histone. *Curr. Biol.* 23 1939–1944. 10.1016/j.cub.2013.07.083 24076245 PMC3796065

[B17] Hansen K. R. Ibarra P. T. Thon G. (2006). Evolutionary-conserved telomere-linked helicase genes of fission yeast are repressed by silencing factors, RNAi components and the telomere-binding protein Taz1. *Nucleic Acids Res.* 34 78–88. 10.1093/nar/gkj415 16407326 PMC1326240

[B18] Henikoff S. Dreesen T. D. (1989). Trans-inactivation of the *Drosophila* brown gene: evidence for transcriptional repression and somatic pairing dependence. *Proc. Natl. Acad. Sci. U.S.A.* 86 6704–6708. 10.1073/pnas.86.17.6704 2505257 PMC297914

[B19] Ingvarsdottir K. Edwards C. Lee M. G. Lee J. S. Schultz D. C. Shilatifard A. (2007). Histone H3 K4 demethylation during activation and attenuation of GAL1 transcription in *Saccharomyces cerevisiae*. *Mol. Cell. Biol.* 27 7856–7864. 10.1128/MCB.00801-07 17875926 PMC2169161

[B20] Kapoor S. Zhu L. Froyd C. Liu T. Rusche L. N. (2015). Regional centromeres in the yeast *Candida lusitaniae* lack pericentromeric heterochromatin. *Proc. Natl. Acad. Sci. U.S.A.* 112 12139–12144. 10.1073/pnas.1508749112 26371315 PMC4593121

[B21] Kaur R. Domergue R. Zupancic M. L. Cormack B. P. (2005). A yeast by any other name: *Candida glabrata* and its interaction with the host. *Curr. Opin. Microbiol.* 8 378–384. 10.1016/j.mib.2005.06.012 15996895

[B22] Ketel C. Wang H. S. W. McClellan M. Bouchonville K. Selmecki A. Lahav T. (2009). Neocentromeres form efficiently at multiple possible loci in *Candida albicans*. *PLoS Genet.* 5:e1000400 10.1371/journal.pgen.1000400 PMC2642679 19266018

[B23] Kouzarides T. (2007). Chromatin modifications and their function. *Cell* 128 693–705. 10.1016/j.cell.2007.02.005 17320507

[B24] Kueng S. Oppikofer M. Gasser S. M. (2013). SIR proteins and the assembly of silent chromatin in budding yeast. *Annu. Rev. Genet.* 47 275–306. 10.1146/annurev-genet-021313-173730 24016189

[B25] Lachner M. Sengupta R. Schotta G. Jenuwein T. (2004). Trilogies of histone lysine methylation as epigenetic landmarks of the eukaryotic genome. *Cold Spring Harb. Symp. Quant. Biol.* 69 209–218. 10.1101/sqb.2004.69.209 16117651

[B26] Merrick C. J. Duraisingh M. T. (2006). Heterochromatin-mediated control of virulence gene expression. *Mol. Microbiol.* 62 612–620. 10.1111/j.1365-2958.2006.05397.x 17076663

[B27] Mishra P. K. Baum M. Carbon J. (2007). Centromere size and position in *Candida albicans* are evolutionarily conserved independent of DNA sequence heterogeneity. *Mol. Genet. Genomics* 278 455–465. 10.1007/s00438-007-0263-8 17588175

[B28] Nonaka N. Kitajima T. Yokobayashi S. Xiao G. Yamamoto M. Grewal S. I. S. (2002). Recruitment of cohesin to heterochromatic regions by Swi6/HP1 in fission yeast. *Nat. Cell Biol.* 4 89–93. 10.1038/ncb739 11780129

[B29] Padmanabhan S. Thakur J. Siddharthan R. Sanyal K. (2008). Rapid evolution of Cse4p-rich centromeric DNA sequences in closely related pathogenic yeasts, *Candida albicans* and *Candida dubliniensis*. *Proc. Natl. Acad. Sci. U.S.A.* 105 19797–19802. 10.1073/pnas.0809770105 19060206 PMC2604992

[B30] Pidoux A. Mellone B. Allshire R. (2004). Analysis of chromatin in fission yeast. *Methods* 33 252–259. 10.1016/j.ymeth.2003.11.021 15157893

[B31] Raman S. B. Nguyen M. H. Zhang Z. Cheng S. Jia H. Y. Weisner N. (2006). *Candida albicans* SET1 encodes a histone 3 lysine 4 methyltransferase that contributes to the pathogenesis of invasive candidiasis. *Mol. Microbiol.* 60 697–709. 10.1111/j.1365-2958.2006.05121.x 16629671

[B32] Rusche L. N. Kirchmaier A. L. Rine J. (2003). The establishment, inheritance, and function of silenced chromatin in *Saccharomyces cerevisiae*. *Annu. Rev. Biochem.* 72 481–516. 10.1146/annurev.biochem.72.121801.161547 12676793

[B33] Ryu H.-Y. Ahn S. (2014). Yeast histone H3 lysine 4 demethylase Jhd2 regulates mitotic ribosomal DNA condensation. *BMC Biol.* 12:75 10.1186/s12915-014-0075-3 PMC4201760 25248920

[B34] Sanyal K. Baum M. Carbon J. (2004). Centromeric DNA sequences in the pathogenic yeast *Candida albicans* are all different and unique. *Proc. Natl. Acad. Sci. U.S.A.* 101 11374–11379. 10.1073/pnas.0404318101 15272074 PMC509209

[B35] Shang W. H. Hori T. Martins N. M. C. Toyoda A. Misu S. Monma N. (2013). Chromosome engineering allows the efficient isolation of vertebrate neocentromeres. *Dev. Cell* 24 635–648. 10.1016/j.devcel.2013.02.009 23499358 PMC3925796

[B36] Shankaranarayana G. D. Motamedi M. R. Moazed D. Grewal S. I. S. (2003). Sir2 regulates histone H3 lysine 9 methylation and heterochromatin assembly in fission yeast. *Curr. Biol.* 13 1240–1246. 10.1016/S0960-9822(03)00489-5 12867036

[B37] Smith J. S. Boeke J. D. (1997). An unusual form of transcriptional silencing in yeast ribosomal DNA. *Genes Dev.* 11 241–254. 10.1101/gad.11.2.241 9009206

[B38] Strahl B. D. Allis C. D. (2000). The language of covalent histone modifications. *Nature* 403 41–45. 10.1038/47412 10638745

[B39] Thakur J. Sanyal K. (2013). Efficient neocentromere formation is suppressed by gene conversion to maintain centromere function at native physical chromosomal loci in *Candida albicans*. *Genome Res.* 23 638–652. 10.1101/gr.141614.112 23439889 PMC3613581

[B40] Trapnell C. Hendrickson D. G. Sauvageau M. Goff L. Rinn J. L. Pachter L. (2013). Differential analysis of gene regulation at transcript resolution with RNA-seq. *Nat. Biotechnol.* 31 46–53. 10.1038/nbt.2450 23222703 PMC3869392

[B41] Westermann S. Drubin D. G. Barnes G. (2007). Structures and functions of yeast kinetochore complexes. *Annu. Rev. Biochem.* 76 563–591. 10.1146/annurev.biochem.76.052705.160607 17362199

[B42] Wilson R. B. Davis D. Mitchell A. P. (1999). Rapid hypothesis testing with *Candida albicans* through gene disruption with short homology regions. *J. Bacteriol.* 181 1868–1874. 10074081 10.1128/jb.181.6.1868-1874.1999 PMC93587

[B43] Wirén M. Silverstein R. A. Sinha I. Walfridsson J. Lee H.-M. Laurenson P. (2005). Genomewide analysis of nucleosome density histone acetylation and HDAC function in fission yeast. *EMBO J.* 24 2906–2918. 10.1038/sj.emboj.7600758 16079916 PMC1187943

