# Supplementary material for: The Chromatin of Candida albicans Pericentromeres Bears Features of Both Euchromatin and Heterochromatin
Source: Front Microbiol. 2016 May 19;7:759. doi: 10.3389/fmicb.2016.00759 (PMC4871872; doi:10.3389/fmicb.2016.00759)
Supplement: Supplementary file 1 [file Table_1.DOCX]

Supplementary Material

**The chromatin state of *Candida albicans* pericentromeric repeats bears features of both euchromatin and heterochromatin**

**Verónica Freire-Benéitez,** **Robert Jordan Price and Alessia Buscaino***

*** Correspondence:** Alessia Buscaino : A.Buscaino@kent.ac.uk

*Supplementary table 1. Strains used in this study*

| **Strain number** | **Description** | **Genotype** |
| --- | --- | --- |
| Bu_20 | *sir2*Δ/Δ | *ura3Δ::λimm434/ura3Δimm434 his1::hisG/his1::hisG arg4::hisG/arg4::hisG sir2 Δ::HIS1/sir2 Δ::ARG4* |
| Bu_70 | *set1*Δ/Δ | *arg4Δ/arg4Δ his1Δ/his1Δ leu2Δ/leu2Δ URA3/ura3Δ::λimm434 IRO1/iro1Δ::λimm434 set1Δ::C.d.HIS1/set1Δ::C.m.LEU2* |
| Bu_215 | BWP17 | *ura3Δ::λimm434/ura3Δ::λimm434 his1::hisG/his1::hisG arg4::hisG/arg4::hisG* |
| Bu_225 | *peri-CEN5:URA3^+^* | *rpt-CEN5::URA3 ura3Δ::λimm434/ura3Δimm434 HIS1::his1::hisG/his1::hisG ARG4::arg4::hisG/arg4::hisG* |
| Bu_243 | *peri-CEN5:URA3^+^ sir2Δ/Δ* | *ura3Δ::λimm434/ura3Δ::λimm434 his1::hisG/his1::hisG arg4::hisG/arg4::hisG CEN:URA3 sir2Δ::HIS1/sir2Δ::NAT* |
| Bu_380 | *jhd2*Δ/Δ | *ura3Δ::λimm434/ura3Δimm434 his1::hisG/his1::hisG arg4::hisG/arg4::hisG jhd2 Δ::HIS1/jhd2 Δ::ARG4* |
| Bu_419 | *peri-CEN4:URA3^+^* | *rpt-CEN4::URA3 ura3Δ::λimm434/ura3Δimm434 HIS1::his1::hisG/his1::hisG ARG4::arg4::hisG/arg4::hisG* |
| Bu_420 | *peri-CEN7:URA3^+^* | *CEN7::URA3 ura3Δ::λimm434/ura3Δimm434 HIS1::his1::hisG/his1::hisG ARG4::arg4::hisG/arg4::hisG* |

*Supplementary table 2. Primers used in this study*

| **Primer** | **Sequence** | **Figure** | **Description** |
| --- | --- | --- | --- |
| Bu_113 | tttcttccggcgtcagacattttgcagttttctatgggatttatggtgtttgtcgaaaaaaaacaagattGTTTTCCCAGTCACGACGTT | Fig 2B-C | *peri-CEN5:URA3*^+^ |
| Bu_114 | tgtatatcttcgaggaatggcaacctttgcccccctctcgaaaaacaatataaatagagtcaatttctctagtagaggtaaattctttgTGTGGAATTGTGAGCGGATA | Fig 2B-C | *peri-CEN5:URA3^+^* |
| Bu_119 | CCCTCTGCTTGTTCGGATTG | Fig Fig 2B-C 3D, 4C, 5B | Primer to check *peri-CEN5:URA3^+^, p5*: qChip |
| Bu_120 | GGCAAGGAACAAGTCACCAG | Fig 3D, 4C, 5B | *p5*: qChip |
| Bu_130 | gtgtgtatggggttgttgctc | Fig 2B-C | Primer to check *rpt- peri-CEN4:URA3^+,^ peri-CEN5:URA3^+^, peri-CEN7:URA3^+^* |
| Bu_139 | GAGTGAGTGAGTGGAGTAGCG | Fig4C | Primer to check *HIS1/NAT* replacement of *SIR2* |
| Bu_141 | gttgggcagatattaccaatg | Fig 2C | *URA3*,RT-qPCR |
| Bu_152 | CTGGAGAAAATATAACCACGAGTCTAAGTTTCTTTATTATATTGACGTTTCAGTTATTTGAGAGAAATCCTCTAGTAgttttcccagtcacgacgtt | Fig 4C | *sir2Δ/Δ* deletion mutant: *HIS1* |
| Bu_153 | ATATATAAATATATAAATATATATATATAAAAGAATTGAAAAGAAAAACATTAAAGACACCAATATTAATTTAAtgtggaattgtgagcggata | Fig 4C | *sir2Δ/Δ* deletion mutant: *HIS1* |
| Bu_164 | cggtctggtaaatgattgac | Fig 4C, 5C | Primer to check *HIS1* integration |
| Bu_165 | AGTGTGGAAAGAAGAGATGC | Fig 5C | Primer to check *ARG4* integration |
| Bu_174 | CTACGTTTCCATTCAAGCTGTT | Fig 2C, 3B, 3D,3F 4C, 5B, 5C | *Act1*: qChip, RT-qPCR |
| Bu_176 | aaactgtaaccacgttcagaca | Fig 2C, 3B, 3D,3F 4C, 5B, 5C | *Act1:* qChip, RT-qPCR |
| Bu_179 | ctgtatctataagcagtatcatcc | Fig 4C | Primer to check *NAT* integration |
| Bu_204 | caaattccttatcggatttagc | Fig 2C | *URA3*, RT-qPCR |
| Bu_286 | CTGGAGAAAATATAACCACGAGTCTAAGTTTCTTTATTATATTGACGTTTCAGTTATTTGAGAGAAATCCTCTAGTAgtaaaacgacggccagtgaattc | Fig 4C | *sir2Δ/Δ* deletion mutant: *NAT* |
| Bu_287 | ATATATAAATATATAAATATATATATATAAAAGAATTGAAAAGAAAAACATTAAAGACACCAATATTAATTTAAtgcatcaattgacgttgatacca | Fig 4C | *sir2Δ/Δ* deletion mutant: *NAT* |
| Bu_466 | TATATTTATTGTGGTTGAAATTTTATATACGTCAAAGATTGCCAAACGTTTGCTGTACACCAAGGTATTTCCAAGgttttcccagtcacgacgtt | Fig5C | *sir2Δ/Δ* deletion mutant: *HIS1/ARG4* |
| Bu_467 | CGTGCTCATAATTAAATGAGAGACATCACTATGCTAATGTCGCAAAAAAACTGTATACATTTCACTACTACACATtgtggaattgtgagcggata | Fig5C | *sir2Δ/Δ* deletion mutant: *HIS1/ARG4* |
| Bu_468 | GATATAGTTATCCCTGGCTA | Fig5C | Primer to check *HIS1/ARG4* replacement of *JHD2* |
| Bu_517 | CCTCCTGTCAACGACCAAC | Fig 3B | *p2*: qChip |
| Bu_518 | AACAGCGGTGGTATAGACGT | Fig 3B | *p2*: qChip |
| Bu_553 | AGAACTCAATGGCACGACTTAAACCCACAACAAAAAGACAATTGAATAAATGATCCTCCTGTCAACGACCAACGTTTTCCCAGTCACGACGTT | Fig 2B-C | *peri-CEN4:URA3^+^* |
| Bu_554 | CTTGAGTTCCGGTGGTCCCTATTTATATTGGTGACATTTCTCTTGGTGATATTTGTGAAGTTACAAATTAAGGTGTGGAATTGTGAGCGGATA | Fig 2B-C | *peri-CEN4:URA3^+^* |
| Bu_555 | GTCAAAGAGTATTACGAATATAAGGAAAAAACTCACGTATTATTCTACAGTAAGCCGAAACAGAACAATAGTGTTTTCCCAGTCACGACGTT | Fig 2B-C | *peri-CEN7:URA3^+^* |
| Bu_556 | AATTACACTAATTGTGACTTAGACAACAGTATACAGCAAGATTGCTAAACTCTATTCATAGTTTTCATCTAGCTGTGGAATTGTGAGCGGATA | Fig 2B-C | *peri-CEN7:URA3^+^* |
| Bu_557 | CTCCCAGTTCCAACACAATTC | Fig 2B-C | Primer to check *peri-CEN4:URA3^+^* |
| Bu_558 | CTGGTCCTCAAGTTGATGTGA | Fig 2B-C | Primer to check peri-*CEN7:URA3^+^* |
| Bu_559 | CATGAAGCGTTCACATGGT | Fig 3F | *p7*: qChip |
| Bu_560 | CGACAGTGTCAGGTATGCTTAG | Fig 3F | *p7*: qChip |
| Bu_561 | TGGAGATTCCTTACGATGGTA | Fig 3F | *p8*: qChip |
| Bu_562 | AGATCACAGCCGACTTCAGT | Fig 3F | *p8*: qChip |
| Bu_563 | GCACCTTGAGCTTGGAGTT | Fig 3F | *p9*:qChip |
| Bu_564 | TCAGGTAATGAATTCAGTGGAG | Fig 3F | *p9*:qChip |
| Bu_565 | AGGCAACCGAAAGAGCTTC | Fig 3B | *p1*: qChip |
| Bu_566 | TAGATGGTAGAGTTCCTCCTGC | Fig 3B | *p1*: qChip |
| Bu_567 | GTCATTAGGATATCGGGACACC | Fig 3B | *p3*:qChip |
| Bu_568 | TCTATGTACAGCAGTAAGGGGTG | Fig 3B | *p3*:qChip |
| Bu_569 | cggccatcattgcactatta | Fig 3D | *p4*: qChip |
| Bu_570 | ctggtgacgatgtcactatgg | Fig 3D | *p4*: qChip |
| Bu_571 | agcttggcatccgacttatt | Fig 3D | *p6*:qChip |
| Bu_572 | ggttccaccgtatatgaagc | Fig 3D | *p6*:qChip |

*Supplementary table 3. Plasmids used in this study*

| **Plasmid** | **Description** |
| --- | --- |
| pGEMURA3 | *URA3* integration products (Wilson et al., 1999) |
| pGEMHIS1 | *HIS1* substitution products (Wilson et al., 1999)  (Wilson et al, 1999) |
| pHA_NAT | *NAT* substitution products (Gerami-nejad et al., 2012) |

**References**

Gerami-nejad, M., Forche, A., Mcclellan, M., and Berman, J. (2012). Analysis of protein function in clinical *C . albicans* isolates. 5314. doi:10.1002/yea.

Wilson, R. B., Davis, D., and Mitchell, A. P. (1999). Rapid Hypothesis Testing with *Candida albicans* through Gene Disruption with Short Homology Regions *J. Bacteriol* 181, 1868-74

**
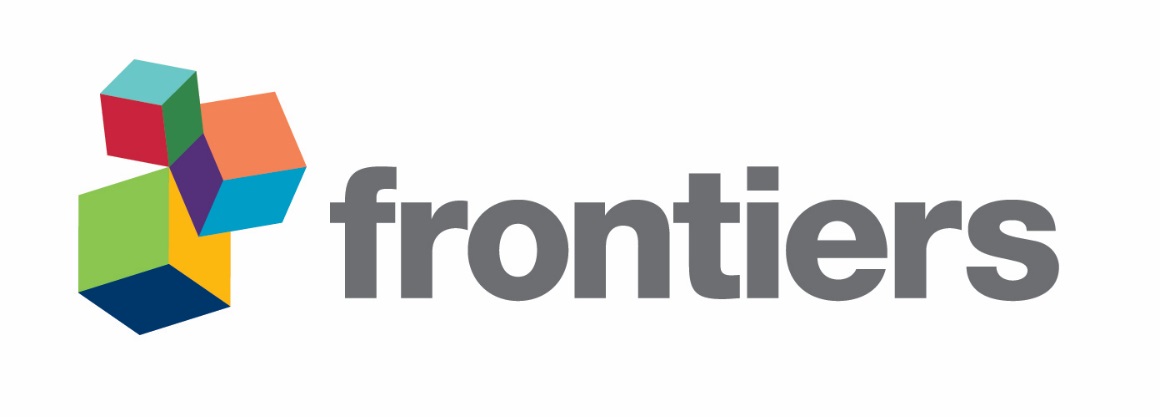
**
